# Question of Morals

**Published:** 1876-02

**Authors:** 


### QUESTION OF MORALS.

SOME TIME since we mentioned, that if some strong-scented cheese was mixed with an equal quantity of pulverized *squills*, and placed where rats and mice could have access to it, they would devour it ravenously, and would soon disappear from the premises.

Quick-lime, or *saleratus*, scattered over the bottom and sides of "holes" through which they pass, will also drive them away, as it "eats" off their skin and hair. Smearing their holes with tar, or catching one, dipping it in a vessel of tar, the head excepted, and then letting it go, is a plan of rat-riddance. But the question is: Ought I to drive my rats into my neighbor's premises? If any one will take the pains to catch a rat, and look it in the face, he will find himself speedily disarmed of his prejudice against the little creature; for there is a sprightliness, a cunning, and withal, a good-naturedness in the expression of a rat's eye, which commands our sympathies. Ought we to kill any creature not necessary for food? Moles have often been ruthlessly pursued; but it is now known that they are harmless to a garden, and preserve it from a variety of depredators.

A humane man convinced a rabid bird-killer of his injustice, by shooting one before his eyes, and, on opening the receptacle for food, a very few grains of wheat were found, but a very large number of worms or weavil.

Bodily activity and bodily health are inseparable. The whole animal creation is stimulated by hunger to an incessant activity of body, and a ceaseless vigilance of the less material part; but that vigilance and activity are expended in extirpating, in many instances, myriads of insects, troublesome to man, and destructive of that which pertains to his support. Some animals are scavengers, whose place could not be supplied by any amount of money or ingenuity that men could bring to bear on the same point. Let us, then, at least inquire, before taking away the life of even a worm—

Is it necessary to the general good or happiness of the great world of living things? Ought we to destroy those creatures which really befriend us? And how boundless is the beneficence of that Providence which makes birds, and four-footed beasts, and creeping things, contribute to the well-being of man, in the performance of a work which is both healthful and pleasureable to themselves? And as to the weaker, that supply food for the stronger, their destruction is a penalty for their want of alertness. Without care, man himself would perish. Without care, watchfulness, birds, and fishes, and animals, and insects, whether in air, on earth or under it, would also perish. "Watch and work" is imperative on all that breathe the breath of life, throughout the universe of the Almighty. "*If any would not* WORK, *neither should he eat*," in the sense of bodily activity, is a requirement of all life: it must be met by the brutes which perish, as well as by man, who is but "*a little lower than the angels*."

